# Experimental Analysis of Kerf Taper Angle in Cutting Process of Sugar Palm Fiber Reinforced Unsaturated Polyester Composites with Laser Beam and Abrasive Water Jet Cutting Technologies

**DOI:** 10.3390/polym13152543

**Published:** 2021-07-31

**Authors:** Fathi Masoud, S. M. Sapuan, Mohd Khairol Anuar Mohd Ariffin, Y. Nukman, Emin Bayraktar

**Affiliations:** 1 Department of Mechanical and Manufacturing Engineering, University Putra Malaysia, Serdang 43400, Selangor, Malaysia; fathimasoud77@gmail.com (F.M.); khairol@upm.edu.my (M.K.A.M.A.); 2 Laboratory of Biocomposite Technology, Institute of Tropical Forestry and Forest Products (INTROP), University Putra Malaysia, Serdang 43400, Selangor, Malaysia; 3 Department of Mechanical Engineering, University of Malaya, Kuala Lumpur 50603, Selangor, Malaysia; nukman@um.edu.my; 4 ISAE-SUPMECA-School of Mechanical and Manufacturing Engineering, 3 Rue Fernand Hainaut, Saint Ouen, 93400 Paris, France

**Keywords:** laser cutting, abrasive waterjet, natural fiber, composite, kerf taper angle

## Abstract

In this research, the effect of processing input parameters on the kerf taper angle response of three various material thicknesses of sugar palm fiber reinforced unsaturated polyester composite was investigated as an output parameter from abrasive waterjet and laser beam cutting techniques. The main purpose of the study is to obtain data that includes the optimum input parameters in cutting the composite utilizing these two unconventional techniques to avoid some defects that arise when using traditional cutting methods for cutting the composites, and then make a comparison to determine which is the most appropriate technique regarding the kerf taper angle response that is desired to be reduced. In the laser beam cutting process, traverse speed, laser power, and assist gas pressure were selected as the variable input parameters to optimize the kerf taper angle. While the water pressure, traverse speed, and stand-off-distance were the input variable parameters in the case of waterjet cutting process, with fixing of all the other input parameters in both cutting techniques. The levels of the input parameters that provide the optimal response of the kerf taper angle were determined using Taguchi’s approach, and the significance of input parameters was determined by computing the max–min variance of the average of the signal to-noise ratio (S/N) for each parameter. The contribution of each input processing parameter to the effects on kerf taper angle was determined using analysis of variation (ANOVA). Compared with the results that were extrapolated in the previous studies, both processes achieved acceptable results in terms of the response of the kerf taper angle, noting that the average values produced from the laser cutting process are much lower than those resulting from the waterjet cutting process, which gives an advantage to the laser cutting technique.

## 1. Introduction

Natural fibers have been primarily viewed as waste and remnants until recently, as they were not efficiently exploited. However, its use is spreading due to the advantages of natural fibers that made them to be an acceptable alternative to synthetic fibers in many applications, especially considering the property of natural decomposition of natural fibers, which makes them environmentally friendly materials [1,2,3,4,5], in addition to that they are extracted from renewable resources that require no energy consumption to produce them, unlike the synthetic fibers production processes. They also have certain other advantages, such as low density, low cost, enhanced recovery, and flexibility [6,7]. Owing to the mentioned benefits, natural fibers have attracted a lot of attention in the advanced polymeric composites field for a variety of engineering applications as a reinforcement material for a broad spectrum of matrices [8,9]. Although composites are formed close to near-net shape, final processes such as drilling, cutting, trimming, and profiling are still required [10,11,12,13,14]. Due to the cutting forces associated with conventional cutting methods and the heterogeneous nature of composites, in addition to specimen fixing that requires a relatively large clamping force, several serious defects appear with the application of traditional cutting techniques in the composites cutting processes, such as, material damage, poor surface quality, delamination, fiber fraying, and dimensional instability [15,16,17,18,19,20]. In order to avert these flaws, non-traditional techniques were considered [12]. Laser beam machining (LBM) and abrasive waterjet machining (AWJM) are the most prominent unconventional technologies utilized in cutting composites due to their high efficiency and productivity [1,9,12,21,22]. In this context, the current study investigates and analyzes the influence of significant input parameters on the kerf taper angle response in cutting three different material thicknesses (2, 4, and 6 mm) of sugar palm fiber reinforced unsaturated polyester (SPF-UPE) composite cut with Laser beam and abrasive waterjet cutting techniques. Sugar palm (Arenga pinnata) that widely spread in South Asia and Southeast Asia, is one of the most versatile palm species since practically every component of the tree can be exploited with palm sap being the most significant product. One of the important product extracted from sugar palm is its fiber, which is characterized a high resistance to sea water and durability, which has traditionally made it a main raw material in the ship ropes production, sugar palm fibers are also distinguished with relatively high tensile strength and thermal resistance, in addition to, that sugar palm fibers are effortless, as they do not need secondary processes, such as mechanical decorticating process to yield them or water retting [23]. One of the most important uses of sugar palm fibers is their employment as a reinforcement material for many polymers, as sugar palm fiber-reinforced polymer composites showed good mechanical, chemical, and thermal properties in many studies that have been conducted on [21,24,25,26,27,28,29]. As one of the most interesting composite materials reinforced with sugar palm fibers is the sugar palm fibers reinforced unsaturated polyester (SPF-UPE), on which several studies have been conducted regarding the evaluation of its physical and chemical properties [23,29,30,31]. There are limited studies on the production and properties of natural fiber reinforced polymers that are relevant to machinability and quality of cut. Despite the fact that there are dozens of relevant studies for synthetic fiber composites, natural fiber reinforced polymers have different responses to cutting processes [32,33]. According to the literature review, there are not enough studies covering required data related to cutting natural fiber composites in general, and there is no study conducted on the material under study related to cutting it with unconventional cutting techniques, so this study was conducted in order to provide sufficient data related to improving the kerf taper angle as one of important output parameters resulting from laser beam and abrasive water jet cutting processes. The data provided in this research would contribute to the exploitation of natural fiber composites, especially the material under study in various applications, such as automobiles, aerospace, construction industries, marine applications, packaging, sporting products, and electronic industries applications. This research covers a good range of material thicknesses in contrast to most of the previous studies that were conducted on natural fiber composites machined using unconventional techniques, which were limited to only one material thickness [9], which makes them difficult to be generalized to different material thicknesses, that may contribute to the limitation of their results in terms of importance. In both cutting processes, the input parameters that have the greatest impact on the kerf taper angle response were selected as variable parameters with three levels of values, while the rest of the input parameters that had no discernible effect on the kerf taper angle were fixed. Nugroho et al. [34] investigated the influence of laser power, traverse speed, gas pressure, and nozzle distance on the kerf properties of agel leaf fiber reinforce unsaturated polyester cut with CO_2_ laser technique, as they reported that the gas pressure ranked first as the most influencing input parameter, followed by cutting speed, laser power, and nozzle distance, respectively. Although previous studies conducted on cutting natural fiber reinforced polymers cut by laser beam technique are limited, as Fathi Masoud et al. [9] reported, but they covered a number of studies in this field and concluded that laser power, traverse speed, and assist gas pressure had the largest effect on the kerf taper angle in the laser beam cutting technique, hence they were chosen as the variable input parameters in this study, while other input parameters, such as focal length, nozzle diameter, and nozzle stand-off distance, did not show a significant effect on kerf taper angle, hence they were kept fixed. In abrasive waterjet cutting process, the selected input parameters were traverse speed, water pressure, and stand-off-distance as they showed the most significant influence on kerf taper angle response, as Fathi Masoud et al. [9], Arumuga Prabu et al. [1], Kalirasu et al. [35], and Jani et al. [16] reported, while the other input parameters like abrasive grain size, nozzle diameter, and impact angle remained constant. Taguchi statistical method was used to determine the optimum input parameters that produce the best response of kerf taper angle in both cutting techniques, and the analysis of variance technique (ANOVA) is used to evaluate the extent to which each parameter contributes to the kerf taper angle response. The significance of this study comes in the fact that it provides crucial information for the process of cutting SPF-UPE composite using laser beam and abrasive waterjet machining technologies, which will help to minimize defects caused by traditional cutting techniques. In contrast to most previous studies, which used low levels of some input parameters, this study covers a wide range of input parameters values. Furthermore, unlike most other research that focused on one specimen thickness, the current study conducted on three various material thicknesses, making its results generalizable to other composites, especially those similar to SPF-UPE in composition and properties.

## 2. Materials and Methods

### 2.1. Fabrication of Composite

**Figure 1:**
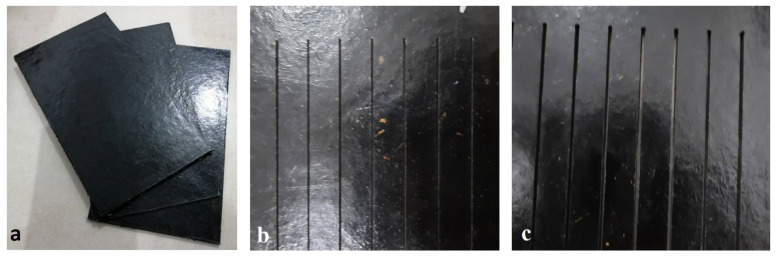
(**a**) The composite specimens before cutting processes. (**b**) Specimen cut with Laser beam cutting technology. (**c**) Specimen cut with abrasive waterjet cutting technology.

Sugar palm fiber reinforced unsaturated polyester (SPF-UPE) composite was used for the research. Sugar palm fibers (SPFs) were cleaned with pure water, dried by hot air, and then treated with 0.25 M/L NaOH with one-hour immersion duration, as this treatment demonstrated good improvement in the mechanical and physical properties of SPF [23,36]. The fibers have been cut manually with lengths from 5 to 10 mm (average aspect ratio 25). The matrix used is unsaturated polyester (UPE) with fiber loading by 30%, as this fiber content showed good mechanical and physical properties [24,37,38]. Three molds with three different depths were utilized to make three different types of specimens with thicknesses of 2, 4, and 6 mm and lengths of 210 mm and widths of 120 mm. The hand lay-up technique was used to perform the composite specimens. The molds were subsequently disassembled and the specimens were removed after 24 h of being covered with a 40 kg weight. Figure 1 shows the produced composite specimens before and after cutting processes.

### 2.2. Experimental Setup

Laser beam cutting experiments were carried out using CO_2_ laser cutting machine (AMADA FO 3015 M2 NT) with a CNC worktable with a maximum power of 4000 W in 1500 Hz pulsed mode. The laser beam was focused on the top surface of the material using a 7.5” focal length lens, the nozzle diameter was 2 mm, the nozzle stand-off distance was 1.5 mm, and air was utilized as the assist gas. Traverse speed, assist gas pressure, and laser power were taken as the input parameters as they demonstrated a significant influence on the kerf taper angle [9,34,39]. Other parameters, such as nozzle diameter, focal length, and nozzle stand-off distance, were kept constant. The various input parameter values were used to investigate three different material thicknesses of 2, 4, and 6 mm. Abrasive water jet cutting experiments were carried out using Flow Mach2 1313B CNC Waterjet machine with operating water pressure up to 60 K psi and Traverse speed up to 10 m/min, 80 mesh (177 microns) garnet abrasive size was used for all of experiments as it gave the best results based on previous studies. The diameter of the nozzle was 1 mm and impact angle was 90°. Traverse speed, water pressure, and stand-off-distance were chosen as the input parameters, as they showed a significant influence on the kerf taper angle [1,9,16,35]. With varied input parameter levels, three distinct material thicknesses of 2, 4, and 6 mm were examined under abrasive water jet machining conditions.

### 2.3. Cutting Parameters Selection

Full thru cutting parameters (FTC) were estimated in the laser beam cutting process for four various values of laser power, namely 100, 1000, 2000, and 3000 W, with a fixed assist gas pressure of 2 bar, and then adjusting the traverse speed until the cut is full thru. This procedure was carried out for all thicknesses. As a result, for each thickness, four groups of parameters were obtained. Cases in which no full cut occurred under any traverse speed value were excluded. Three values have been taken for each parameter in which the full thru cut was obtained. Then, nine cuts with 60 mm length were made at the different levels for the input parameters, based on L9 Taguchi array. Specimens that demonstrated damage, high heat Affected zone (HAZ) and irregular kerf at the cutting zone as results of high laser power, were excluded and the experiments that gave minimal productivity due to low cutting speeds were also excluded. The specimens with regular kerfs, no observed damages and low values of the HAZ were selected for study and optimization. Table 1, Table 2 and Table 3 show the levels of the input parameter for every thickness in laser beam cutting process.

In the abrasive water jet cutting process, the chosen input parameters were water pressure, traverse speed, and stand-off-distance, as they showed the most significant influence on kerf taper angle response [1,9,16,35]. The other parameters such as nozzle diameter, abrasive grain size, and impact angle were kept constant. Abrasive waterjet cutting technology has a great potential to cut various hard and thick materials, so sugar palm fiber reinforced unsaturated polyester (SPF-UPE) is considered a soft material compared to water cutting capabilities, and then it is possible to obtain a full thru cut at low water pressures (100–200) MPa at relatively large traverse speeds. In spite of this fact, clear defects appeared on the specimens at low water pressures and low traverse speeds such as damages and cracks. Incomplete cutting and pull out of the fibers is another type of defect that appeared in the kerf zone at the same conditions of water pressure and traverse speed. Additionally, the large extension of the cutting area was one of the most prominent defects associated with the application of low pressures and high traverse speeds, which also cause uneven cut. Therefore, all parameter values that showed the aforementioned defects were excluded. The best cuts were at relatively high water pressures from 300 to 340 MPa, with corresponding traverse speeds for each material thickness. Thus, nine cuts with 60 mm length were made at the different levels for the input parameters. Table 4, Table 5 and Table 6 show the levels of parameters selected for study and optimization for each material thickness.

### 2.4. Kerf Taper Angle Measurement

Kerf taper angle can be calculated by measuring the top and bottom of kerf width and then applying it to Equation (1) [1,16,35,40], where θ° is kerf taper angle, (Tw) is top kerf width, (Bw) is bottom kerf width, and (t) is material thickness. For the purpose of measuring top and bottom kerf width a reflected industrial microscope OLYMPUS BX51M system with Olympus Stream Essentials image analysis software was used, and a reading every 5 mm has been taken along the cut and then estimating the kerf taper angle and taking the average value.

(1) $$\theta^{{}^{\circ}}=\tan^{-1}\left(\frac{\mathrm{Tw}-\mathrm{Bw}}{2t}\right)$$

### 2.5. Optimization Methods

The Taguchi method’s design of experiments (DOE) was used to investigate the influence of chosen input parameters on the kerf taper angle, where the estimated average values of kerf taper angle were analyzed using the signal-to-noise ratio (S/N) small-is-better calculation to define the desired parameters that produce the smallest kerf taper angle and to identify a significant rank for every parameter. The contribution of each input parameter to the kerf taper angle property was determined using the analysis of variance (ANOVA) method. Minitab software was used to perform all statistical calculations.

## 3. Results and Discussion

### 3.1. Laser Beam Cutting Process

**Figure 2:**
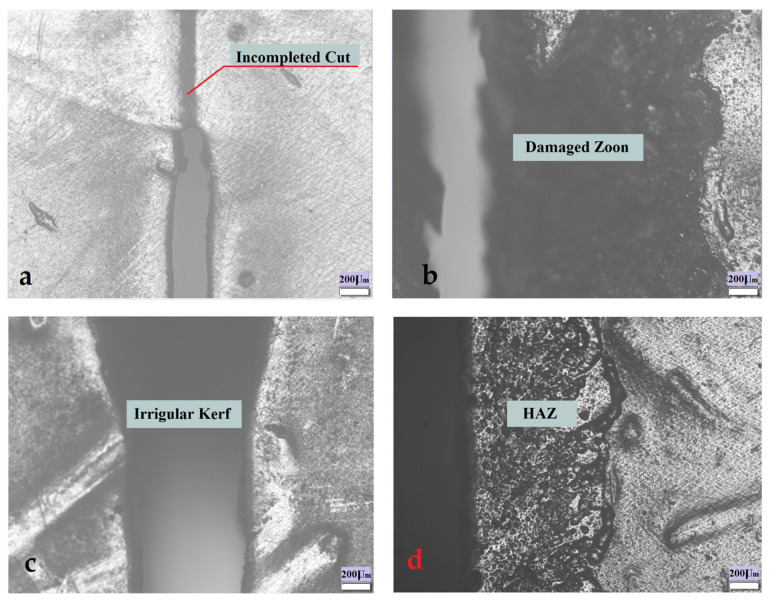
(**a**) Incomplete cut. (**b**) Damage at the cutting zone. (**c**) Irregular kerf. (**d**) High extension of the heat-affected zone (HAZ).

The ranges of input parameters that produced the defects shown in Figure 2 have been excluded, and the parameters in Table 1, Table 2 and Table 3 that gives good observed quality of cutting zone, have been considered for optimizing kerf taper angle property of sugar palm fiber reinforced unsaturated polyester cut with laser beam cutting technique.

**Figure 3:**
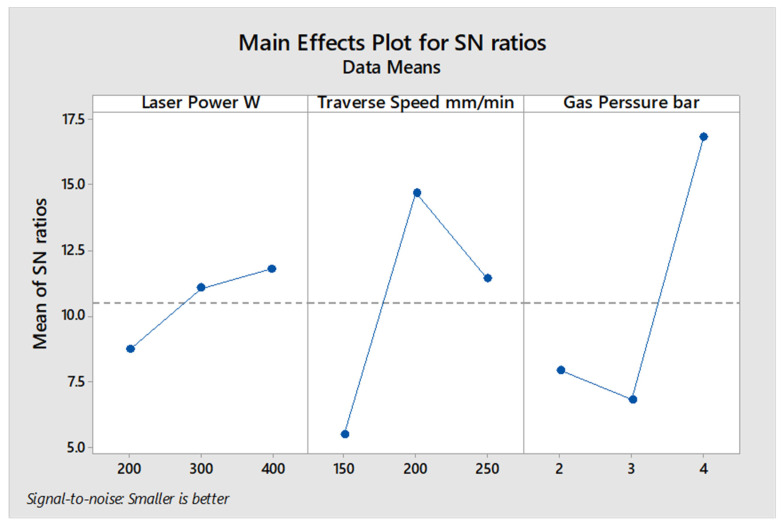
S/N ratio average value of input cutting parameters of the 2 mm thickness of sugar palm fiber reinforced unsaturated polyester (SPF-UPE) composite cut by laser beam cutting process.

Based on measured response of the kerf taper angle in Table 7 for 2 mm material thickness, the average of S/N ratio for every input parameter was estimated and represented in Figure 3. At these conditions of input parameters values, the high gas pressure and laser power and medium traverse speed produce the optimum desired response of kerf taper angle. According to the max–min variation of S/N ratio that was calculated in Table 8, the significance of input parameters can be defined, as gas pressure ranked first as the most significant factor affecting the kerf taper angle, and traverse speed and laser power came second and third, respectively. Regarding to the ANOVA results that shown in Table 9, the contributions of the input parameters were 34.44% for gas pressure, 25.19% for traverse speed, and 2.93% for laser power. Based on Figure 3, 400 W laser power, 200 mm/min traverse speed, and 4 bar assist gas pressure are the optimum input parameters for cutting 2 mm material thickness of the SPF-UPE composite cut using CO_2_ laser beam cutting technology based on the desired response of the kerf taper angle. Based on average S/N ratio represented in Figure 3, and the small contribution of laser power, the medium value can be applied with no large effect because the variation in this parameter did not show an important effect on kerf taper angle response. Girish Dutt Gautam et al. [41] also found that the higher value of assist gas pressure produces the better response of kerf taper angle in cutting Kevlar/basalt fiber-reinforced hybrid composites cut with pulsed Nd:YAG laser cutting technology.

**Figure 4:**
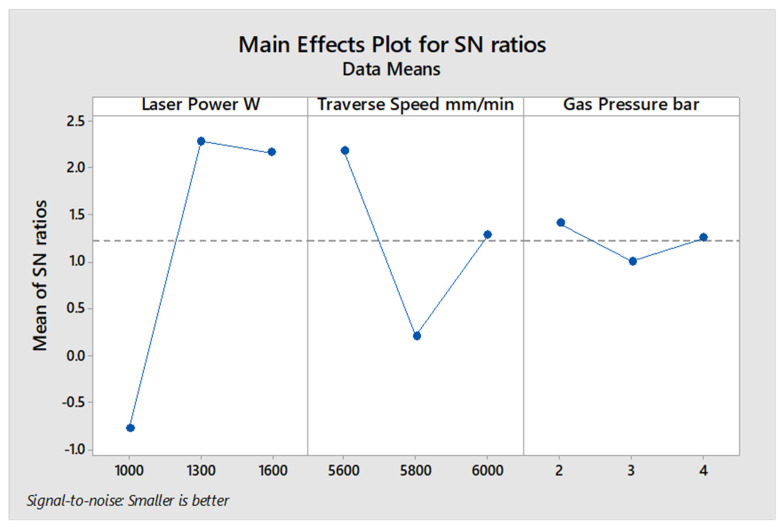
S/N ratio average value of input cutting parameters of the 4 mm thickness of sugar palm fiber reinforced unsaturated polyester (SPF-UPE) composite cut by laser beam cutting process.

Figure 4, illustrates the average values of the S/N ratio for all levels of the controller parameters based on the estimated S/N ratio of the measured kerf taper angle that shown in Table 10 for 4 mm plate thickness. Under these conditions, the medium and upper values of the laser power and minimum traverse speeds gave the best results, while the gas pressure has no significant effect, unlike results recorded at 2 mm material thickness experiment. In Table 11, the level of influence of input parameters were calculated by estimating the max–min variance of the average of the S/N ratio, and based on the ANOVA results in Table 12, laser power ranked first as the most influential parameter on kerf taper angle with contribution of 68.96%, while the Travers speed came second with contribution of 11.80%, and gas pressure did not show an important effect with contribution of 0.94%. Thus, 5600 mm/min traverse speed, 1300 W laser power, and 2 bar assist gas pressure produced the optimum response of the examined processing parameters for 4 mm plate thickness of the SPF-UPE composite cut with CO_2_ laser beam cutting process based on the desired values of kerf taper angle response. The other two levels of assist gas pressure can be applied with no significance effect, because it did not show an important contribution to the response of kerf taper angle. 

**Figure 5:**
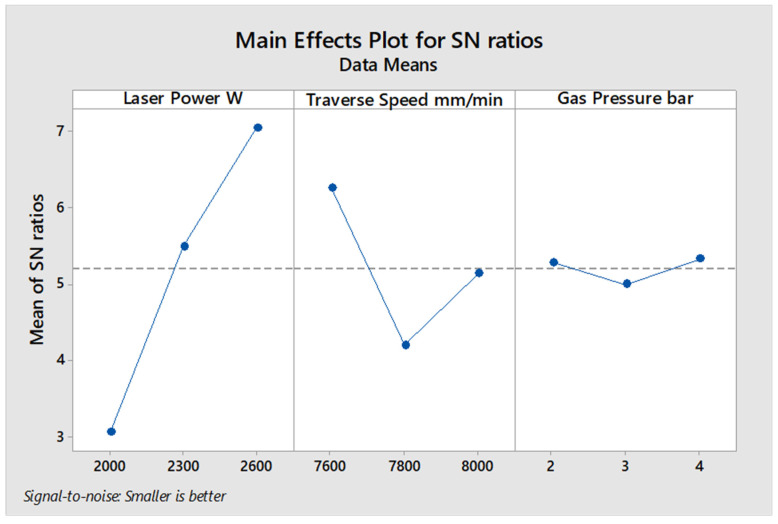
S/N ratio better average value of input cutting parameters of the 6 mm thickness of sugar palm fiber reinforced unsaturated polyester (SPF-UPE) composite cut by laser beam cutting process.

Figure 5 demonstrates the average values of S/N ratio of every level of input parameters in accordance of measured kerf tape angle in Table 13 for 6 mm specimen thickness. The results did not show an important effect of assist gas pressure, while the laser power was the most important parameter in terms of affecting the kerf taper angle, followed by the traverse speed as shown in Table 14. It is observed in Figure 5, the higher values of the laser power and the lower values of the traverse speeds give the best response to the kerf taper angle, and this roughly corresponds to the case of 4 mm material thickness. ANOVA calculations in Table 15 show a significant contribution of the laser power of 81.2% to the effect on the kerf taper angle, while the effect of traverse speed was 13.57%, and the effect of assist gas pressure was very small, not exceeding 2.55%. Thus, 2600 W laser power, 7600 mm/min traverse speed, and 4 bar gas pressure gave the best response of the examined processing parameters for 6 mm plate thickness of the SPF-UPE composite cut with CO_2_ laser beam cutting technology.

**Figure 6:**
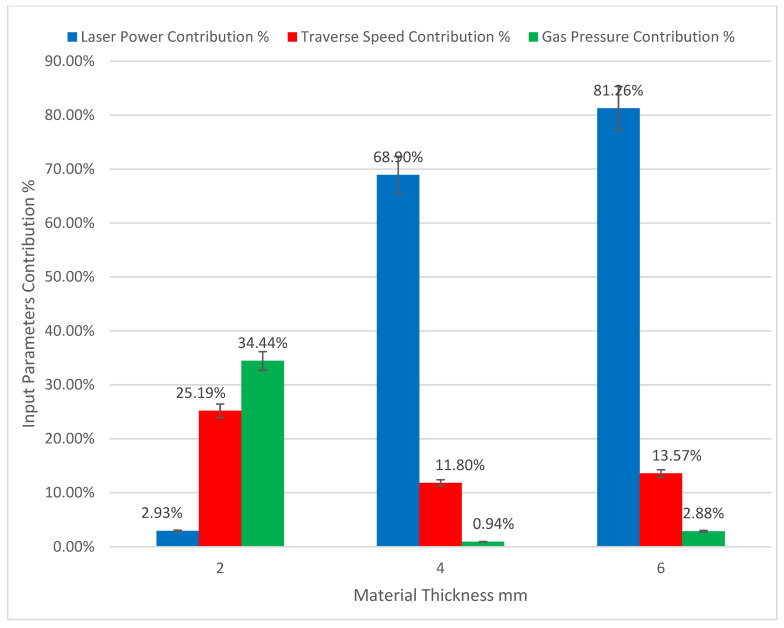
Input parameter contributions to kerf taper angle of the various material thicknesses of SPF-UPE composite cut with laser beam cutting technology.

The contributions of the input parameters to the effect on the kerf taper angle of each material thickness are represented in Figure 6. In small material thicknesses (2 mm), gas pressure takes the largest contribution, as the higher value of assist gas pressure produce the better response of kerf taper angle as this is consistent with what Girish Dutt Gautam et al. [41] found, while traverse speed comes second in terms of effect on kerf taper angle and no significant effect of laser power was observed. This is in contrast to the larger material thicknesses (4 and 6 mm), where the laser power has the largest importance in affecting the kerf taper angle followed by the traverse speed with little effect of assist gas pressure. The best response of taper angle in the case of 4 and 6 mm is obtained with high and medium laser power and low traverse speed, which is consistent with what Ali Solati et al. [42] found, as this study did not demonstrate an importance of assist gas pressure. The high laser power and low traverse speeds allow a large possibility and time to complete the thermal decomposition of the material in the cutting area, and thus completely removing it by assist compressed gas, which may justify obtaining the best kerf properties under these conditions.

### 3.2. Abrasive Water Jet Cutting Process

**Figure 7:**
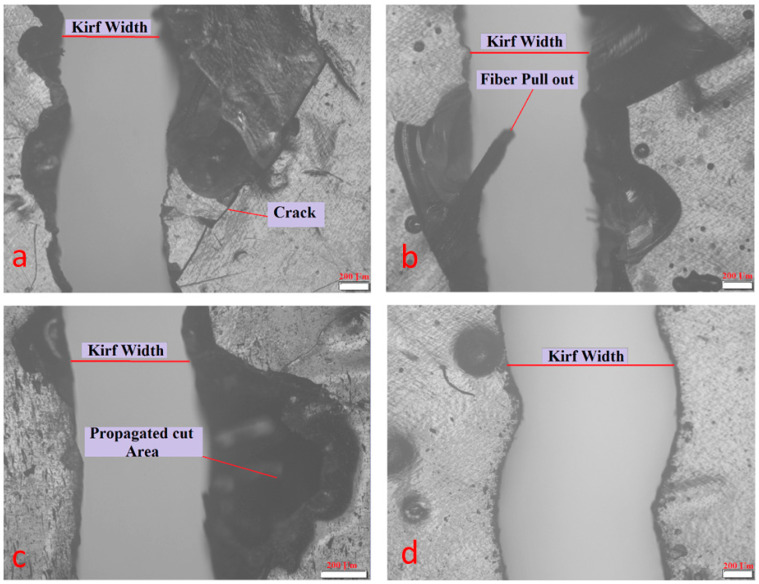
(**a**) Damages and cracks at cutting kerf. (**b**) Incomplete cut and pull out of fibers. (**c**) High propagated cutting area. (**d**) Uneven cut.

For optimizing kerf taper angle response of sugar palm reinforced unsaturated polyester composite cut with abrasive waterjet technology, the ranges of input parameters that produced the flaws shown in Figure 7 were excluded, and the parameters listed in Table 4, Table 5 and Table 6 that gave satisfactory observed cutting zone quality were examined.

**Figure 8:**
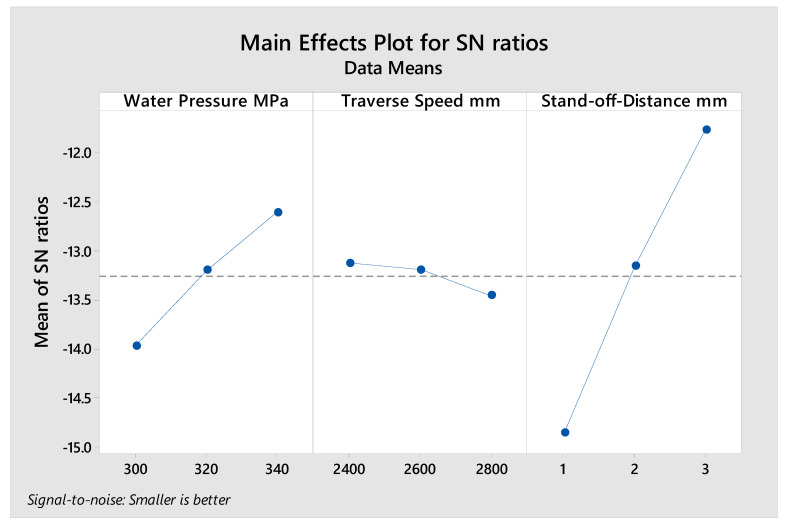
S/N ratio average value of input cutting parameters of the 2 mm thickness of sugar palm fiber reinforced unsaturated polyester (SPF-UPE) composite cut with abrasive water jet technique.

The average of S/N ratio for each input parameter was computed and presented in Figure 8 based on the measured response of the kerf taper angle in Table 16 for 2 mm material thickness cut with abrasive waterjet machining technology. The high stand-off-distance, high water pressure, and low traverse speed produced the optimum desired kerf taper angle response at these input parameter levels. According to the max–min variation of S/N ratio that was calculated in Table 17, the significance of input parameters can be defined, as stand-off-distance ranked first as the most significant factor affecting the kerf taper angle, and water pressure and traverse speed came second and third, respectively. Regarding the ANOVA results shown in Table 18, the contributions of the input parameters were 83.48% for stand-off-distance, 15.70% for water pressure, and 0.81% for traverse speed. Based on Figure 8, 340 Mpa water pressure, 2400 mm/min traverse speed, and 3 mm stand-off-distance are the optimum input parameters for cutting 2 mm material thickness of the SPF-UPE composite cut using abrasive water jet cutting technology based on the desired response of the kerf taper angle. Based on the average S/N ratio represented in Figure 8 and the small contribution of traverse speed, the medium and high values can be applied with no large effect.

**Figure 9:**
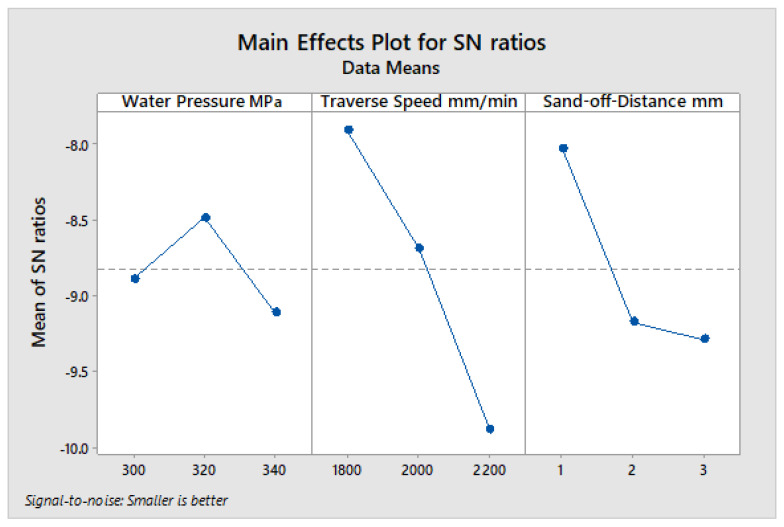
S/N ratio average value of input cutting parameters of the 4 mm thickness of sugar palm fiber reinforced unsaturated polyester (SPF-UPE) composite cut with abrasive water jet technique.

Based on the estimated S/N ratio of the measured kerf taper angle reported in Table 19 for 4 mm plate thickness, Figure 9 shows the average values of the S/N ratio for all levels of the input parameters. Under these conditions, the best responses of kerf taper angle were obtained by applying the low traverse speed, low stand-off-distance, and medium water pressure. In Table 20, the level of influence of input parameters were calculated by estimating the max–min variance of the average of S/N ratio, and based on the ANOVA results in Table 21, traverse speed ranked first as the most influential parameter on kerf taper angle with contribution of 61.20%, while stand-off-distance came second with contribution of 34.17%, and water pressure did not show an important effect with a contribution of 4.33%. Thus, 1800 mm/min traverse speed, 1 mm stand-off-distance, and 320 Mpa water pressure produced the optimum response of the examined processing parameters for 4 mm plate thickness of the SPF-UPE composite cut with waterjet cutting process based on the desired values of kerf taper angle response. Due to the small contribution of the change in water pressure to the effect on kerf taper angle, the lower value of the water pressure can be applied instead of the average value.

**Figure 10:**
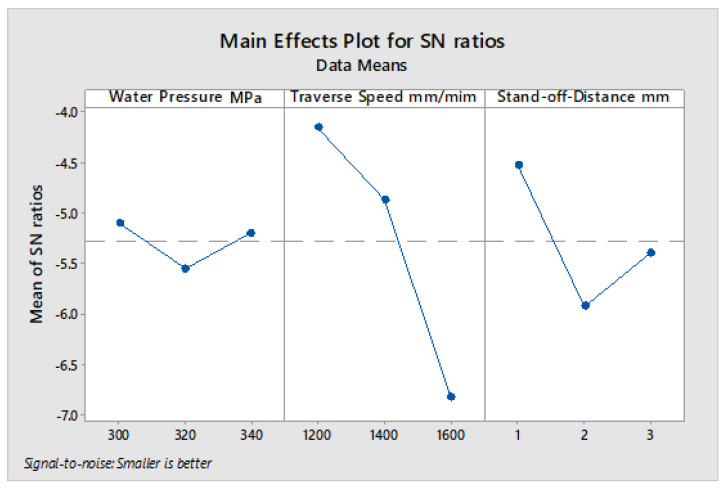
S/N ratio average value of input cutting parameters of the 6 mm thickness of sugar palm fiber reinforced unsaturated polyester (SPF-UPE) composite cut with abrasive water jet technique.

Figure 10 shows the average S/N ratio values for each level of input parameters based on the measured kerf tape angle in Table 22 for a specimen with 6 mm thickness. The results showed that water pressure had no significant effect on the kerf taper angle, whereas traverse speed was the most important factor impacting the kerf taper angle, followed by stand-off-distance as indicated in Table 23 of max-min variance of S/N ratio. As it is seen in Figure 10, the optimal response of kerf taper angle can be obtained with applying the lower value of traverse speed, stand-off-distance, and water pressure, taking into account that the water pressure did not show a significant effect. ANOVA calculations represented in Table 24, show an important contribution of traverse speed of 70.35% to the effect on the kerf taper angle, whereas the effect of stand-off-distance was 18.23%, with a small contribution of water pressure, not exceeding 2.06%. The best response produced by the tested processing parameters for 6 mm plate thickness of the SPF-UPE composite cut with water jet cutting technology was at 1200 mm/min traverse speed, 1 mm stand-off-distance, and 300 Mpa water pressure.

**Figure 11:**
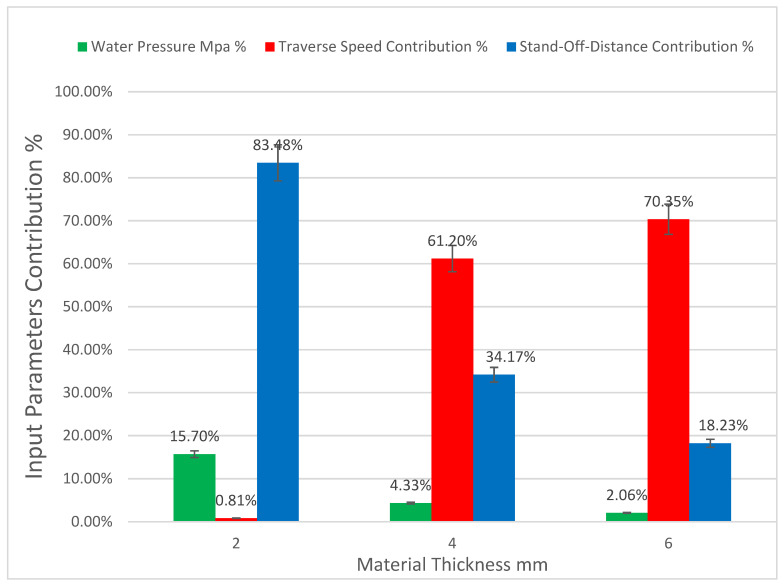
Input parameter contributions to kerf taper angle of the various material thicknesses of SPF-UPE composite cut with waterjet cutting technology.

In the case of 2 mm material, stand-off-distance makes the largest contribution, as illustrated in Figure 11, which shows the contributions of the input parameters to the effect on the kerf taper angle response of each material thickness. The higher level of stand-off-distance (3 mm) gave the optimal response, as it is shown in Figure 7, which is consistent with what Kalirasu et al. [43] found examining various types of natural fiber reinforced unsaturated polyester, except that the stand-off-distance was not of large significance, contrary what was found in this study. In the cases of medium and large material thicknesses, traverse speed has the largest contribution followed by stand-off-distance with small effect of water pressure, as the lower levels of traverse speeds gave the best kerf taper angle response contrary to the result found by Arumuga Prabu et al. [1] that worked on Banana Fiber Reinforced Polyester Composite, but they found that the optimal response can be obtained with applying the lower levels of stand-off-distance similar to what found in this research. In the cases of 4 and 6 mm material thicknesses, better kerf taper angle can be achieved at a low traverse speed, and low standoff distance, which is consistent with most of the studies surveyed by R. K. Thakur et al. [44].

**Figure 12:**
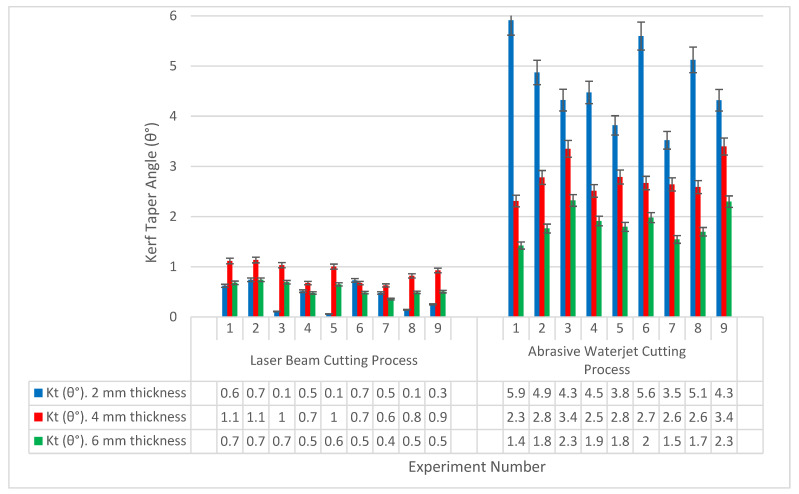
Kerf taper angle (θ°) relative to experiment number and specimen thickness for laser beam and abrasive water jet cutting processes.

A comparison can be drawn between both cutting processes in terms of the kerf taper angle value and its relationship to the thickness of the material. In Figure 12 the kerf taper angle response of every single experiment number for both processes is represented. The figure clearly shows that the values of the kerf taper angle in the case of laser cutting process were much less than in the case of waterjet cutting process, but the abrasive waterjet cutting process produced an acceptable response compared to the results that have been found in some previous researches, such as the studies conducted by Arumuga Prabu et al. [1] and Kalirasu et al. [43]. It is also noted that the lowest kerf taper angle response was recorded at the lowest material thickness in the case of laser cutting process, contrary to the case of abrasive waterjet cutting process in which the largest responses of the kerf taper angle were recorded at the same material thickness, which may be due to the large erosion caused by the waterjet and the abrasive grains. It is also noted that the specimen with the smallest thickness (2 mm) is subjected to a significant amount of vibrations, which may contribute to the growth of the kerf taper angle in the case of abrasive water jet cutting process, meanwhile the other specimens were more rigid under the process conditions, and this may explain the decreasing in the kerf taper angle with the increase in the material thickness. In the case of the laser cutting process, the largest average of kerf taper angle values was recorded at the thicker material (6 mm), which may be because the farther parts of the material along the depth of the cut may be exposed to less amounts of heat, which makes this area less decomposing than the higher areas, where the laser beam is focused at the upper surface of the material, which makes the cut at the top wider than the bottom, leading to an increase of the kerf taper angle values.

## 4. Conclusions

The laser beam and abrasive waterjet cutting processes of sugar palm fiber reinforced unsaturated polyester composite are completed satisfactorily, with the following findings:

- The average of the kerf taper angle in the case of laser beam cutting process was less than in the case of waterjet cutting process, as the value did not exceed 1.034° in the case of laser beam cutting technology, while it ranged from 1.42° to 5.91° in the case of water cutting machining, and this gives an advantage to the laser beam cutting technology.
- No negative kerf taper angles were recorded in both cutting processes, which means that the upper kerf width was wider than the lower kerf width of the specimens in all experiments.
- In laser beam cutting process, assist gas pressure has the largest influence on the kerf taper angle response, followed by traverse speed and laser power, respectively, for 2 mm material thicknesses, meanwhile, laser power took the greatest influence on the kerf taper angle, followed by traverse speed with small contribution of assist gas pressure in the cases of 4 mm and 6 mm specimen thicknesses.
- Optimum input parameters that produced the best response of kerf taper angle in laser cutting process were, 4 bar assist gas pressure, 200 mm/min traverse speed, and 400 W laser power for 2 mm material thickness. In the case of 4 mm material thickness the optimum input parameters were, 1300 W laser power, 5600 mm/min traverse speed, and 2 bar assist pressure, meanwhile the optimum input parameters for 6 mm specimen thickness were, 2600 W laser power, 7600 mm/min traverse speed, and 4 bar assist pressure.
- In waterjet cutting process, stand-off-distance has the largest influence on the kerf taper angle response, followed by water pressure with small contribution of traverse speed, for 2 mm material thicknesses, while traverse speed has the greatest influence on the kerf taper angle, followed by stand-off-distance and water pressure, respectively, in the cases of 4 mm and 6 mm specimen thicknesses.
- Optimum input parameters that gave the best response of kerf taper angle in waterjet cutting technology were 3 mm stand-off-distance, 2400 mm/min traverse speed, and 340 MPa water pressure for 2 mm material thickness. In the case of 4 mm material thickness, the optimum input parameters were 1 mm stand-off-distance, 1800 mm/min traverse speed, and 320 MPa water pressure, while the optimum input parameters for 6 mm specimen thickness were 1 mm stand-off-distance, 1200 mm/min traverse speed, and 300 MPs water pressure.

## Figures and Tables

**Table 1:** Input parameters levels for specimen thickness of 2 mm, in laser beam cutting process.

Parameters	Level 1	Level 2	Level 3
Laser Power (W)	200	300	400
Traverse Speed (mm/min)	150	200	250
Gas Pressure (bar)	2	3	4

**Table 2:** Input parameters levels for specimen thickness of 4 mm, in laser beam cutting process.

Parameters	Level 1	Level 2	Level 3
Laser Power (W)	1000	1300	1600
Traverse Speed (mm/min)	5600	5800	6000
Gas Pressure (bar)	2	3	4

**Table 3:** Input parameters levels for specimen thickness of 6 mm, in laser beam cutting process.

Parameters	Level 1	Level 2	Level 3
Laser Power (W)	2000	2300	2600
Traverse Speed (mm/min)	7600	7800	8000
Gas Pressure (bar)	2	3	4

**Table 4:** Input parameters levels for specimen thickness of 2 mm, in the abrasive waterjet cutting process.

Parameters	Level 1	Level 2	Level 3
Water Pressure (Mpa)	300	320	340
Traverse Speed (mm/min)	2400	2600	2800
Stand-off-Distance (mm)	1	2	3

**Table 5:** Input parameters levels for specimen thickness of 4 mm, in the abrasive waterjet cutting process.

Parameters	Level 1	Level 2	Level 3
Water Pressure (Mpa)	300	320	340
Traverse Speed (mm/min)	1800	2000	2200
Stand-off-Distance (mm)	1	2	3

**Table 6:** Input parameters levels for specimen thickness of 6 mm, in the abrasive waterjet cutting process.

Parameters	Level 1	Level 2	Level 3
Water Pressure (Mpa)	300	320	340
Traverse Speed (mm/min)	1200	1400	1600
Stand-off-Distance (mm)	1	2	3

**Table 7:** L9 array of input parameter levels with measured Kerf taper angle θ° in degrees and calculated signal-to-noise (S/N) ratio based on the Taguchi method for 2 mm specimen thickness.

Ex No:	Laser Power W	Traverse Speed mm/min	Gas Pressure Bar	Kerf Taper Angle Degrees	S/N
1	200	150	2	0.620	4.1522
2	200	200	3	0.740	2.6154
3	200	250	4	0.107	19.4123
4	300	150	3	0.513	5.7977
5	300	200	4	0.059	24.5830
6	300	250	2	0.728	2.7574
7	400	150	4	0.477	6.4296
8	400	200	2	0.143	16.8933
9	400	250	3	0.250	12.0412

**Table 8:** S/N ratio response table of input cutting parameters of the 2 mm thickness off SPF-UPE composite cut by laser beam cutting process.

Level	Laser Power W	Traverse Speed mm/min	Gas Pressure Bar
1	8.727	5.460	7.934
2	11.046	14.697	6.818
3	11.788	11.404	16.808
Delta	3.061	9.237	9.990
Rank	3	2	1

**Table 9:** ANOVA table for kerf taper angle response of input cutting parameters of 2 mm thickness of SPF-UPE composite cut by laser beam cutting process.

Source	DF	Seq SS	Contribution	Adj SS	Adj MS	F-Value	P-Value
Laser Power W	2	0.2028	2.93%	0.2028	0.1014	0.08	0.927
Traverse Speed mm/min	2	1.7431	25.19%	1.7431	0.8715	0.67	0.598
Gas Pressure bar	2	2.3832	34.44%	2.3832	1.1916	0.92	0.521
Error	2	2.5911	37.44%	2.5911	1.2956		
Total	8	6.9202	100.00%				

**Table 10:** L9 array of input parameter levels with measured Kerf taper angle θ° in degrees and calculated signal-to-noise (S/N) ratio based on the Taguchi method for 4 mm specimen thickness.

Ex No:	Laser Power W	Traverse Speed mm/min	Gas Pressure Bar	Kerf Taper Angle Degrees	S/N
1	1000	5600	2	1.116	−0.95328
2	1000	5800	3	1.134	−1.09226
3	1000	6000	4	1.032	−0.27359
4	1300	5600	3	0.674	3.42680
5	1300	5800	4	1.003	−0.02602
6	1300	6000	2	0.674	3.42680
7	1600	5600	4	0.627	4.05465
8	1600	5800	2	0.818	1.74493
9	1600	6000	3	0.925	0.67717

**Table 11:** S/N ratio response table of input cutting parameters of the 4 mm thickness off SPF-UPE composite cut by laser beam cutting process.

Level	Laser Power W	Traverse Speed mm/min	Gas Pressure Bar
1	−0.7730	2.1761	1.4062
2	2.2759	0.2089	1.0039
3	2.1589	1.2768	1.2517
Delta	3.0489	1.9672	0.4022
Rank	1	2	3

**Table 12:** ANOVA table for kerf taper angle response of input cutting parameters of 4 mm thickness of SPF-UPE composite cut by the laser beam cutting process.

Source	DF	Seq SS	Contribution	Adj SS	Adj MS	F-Value	P-Value
Laser Power W	2	0.238035	68.90%	0.238035	0.119018	3.76	0.210
Traverse Speed mm/min	2	0.040779	11.80%	0.040779	0.020390	0.64	0.609
Gas Pressure bar	2	0.003260	0.94%	0.003260	0.001630	0.05	0.951
Error	2	0.063387	18.35%	0.063387	0.031693		
Total	8	0.345461	100.00%				

**Table 13:** L9 array of input parameter levels with measured Kerf taper angle θ° in degrees and calculated signal-to-noise (S/N) ratio based on the Taguchi method for 6 mm specimen thickness.

Ex No:	Laser Power W	Traverse Speed mm/min	Gas Pressure Bar	Kerf Taper Angle Degrees	S/N
1	2000	7600	2	0.680	3.34982
2	2000	7800	3	0.740	2.61537
3	2000	8000	4	0.692	3.19788
4	2300	7600	3	0.477	6.42963
5	2300	7800	4	0.648	3.76850
6	2300	8000	2	0.485	6.28517
7	2600	7600	4	0.354	9.01993
8	2600	7800	2	0.489	6.21382
9	2600	8000	3	0.505	5.93417

**Table 14:** S/N ratio response table of input cutting parameters of the 6 mm thickness off SPF-UPE composite cut by the laser beam cutting process.

Level	Laser Power W	Traverse Speed mm/min	Gas Pressure Bar
1	3.054	6.266	5.283
2	5.494	4.199	4.993
3	7.056	5.139	5.329
Delta	4.002	2.067	0.336
Rank	1	2	3

**Table 15:** ANOVA table for kerf taper angle response of input cutting parameters of 6 mm thickness of SPF-UPE composite cut by the laser beam cutting process.

Source	DF	Seq SS	Contribution	Adj SS	Adj MS	F-Value	P-Value
Laser Power W	2	0.187757	81.26%	0.187757	0.093878	35.48	0.027
Traverse Speed mm/min	2	0.031353	13.57%	0.031353	0.015676	5.92	0.144
Gas Pressure bar	2	0.006643	2.88%	0.006643	0.003321	1.26	0.443
Error	2	0.005293	2.29%	0.005293	0.002646		
Total	8	0.231045	100.00%				

**Table 16:** L9 array of input parameter levels with measured Kerf taper angle θ° in degrees and calculated signal-to-noise (S/N) ratio based on the Taguchi method for 2 mm specimen thickness cut with abrasive water jet technique.

Ex No:	Water Pressure MPa	Traverse Speed mm	Stand-Off-Distance mm	Kerf Taper Angle θ°	SNRA1
1	300	2400	1	5.911	−15.4332
2	300	2600	2	4.872	−13.7541
3	300	2800	3	4.321	−12.7117
4	320	2400	2	4.474	−13.0139
5	320	2600	3	3.818	−11.6367
6	320	2800	1	5.597	−14.9591
7	340	2400	3	3.521	−10.9333
8	340	2600	1	5.123	−14.1905
9	340	2800	2	4.317	−12.7036

**Table 17:** S/N ratio response table of input cutting parameters of the 2 mm thickness off SPF-UPE composite cut with abrasive water jet technique.

Level	Water Pressure MPa	Traverse Speed mm	Stand-Off-Distance mm
1	−13.97	−13.13	−14.86
2	−13.20	−13.19	−13.16
3	−12.61	−13.46	−11.76
Delta	1.36	0.33	3.10
Rank	2	3	1

**Table 18:** ANOVA table for kerf taper angle response of input cutting parameters of 2 mm thickness of SPF-UPE composite cut with abrasive water jet technique.

Source	DF	Seq SS	Contribution	Adj SS	Adj MS	F-Value	P-Value
Water Pressure MPa	2	0.041841	15.70%	0.041841	0.020920	1371.69	0.001
Traverse Speed mm/min	2	0.002156	0.81%	0.002156	0.001078	70.68	0.014
Stand-Off-Distance mm	2	0.222526	83.48%	0.222526	0.111263	7295.20	0.000
Error	2	0.000031	0.01%	0.000031	0.000015		
Total	8	0.266553	100.00%				

**Table 19:** L9 array of input parameter levels with measured Kerf taper angle θ° in degrees and calculated signal-to-noise (S/N) ratio based on the Taguchi method for 4 mm specimen thickness cut with abrasive water jet technique.

Ex No:	Water Pressure MPa	Traverse Speed mm	Stand-Off-Distance mm	Kerf Taper Angle θ°	SNRA1
1	300	1800	1	2.310	−7.2722
2	300	2000	2	2.780	−8.8809
3	300	2200	3	3.350	−10.5009
4	320	1800	2	2.512	−8.0004
5	320	2000	3	2.790	−8.9121
6	320	2200	1	2.670	−8.5302
7	340	1800	3	2.642	−8.4387
8	340	2000	1	2.589	−8.2626
9	340	2200	2	3.398	−10.6245

**Table 20:** S/N ratio response table of input cutting parameters of the 4 mm thickness off SPF-UPE composite cut with abrasive water jet technique.

Level	Water Pressure MPa	Traverse Speed mm	Stand-Off-Distance mm
1	−8.885	−7.904	−8.022
2	−8.481	−8.685	−9.169
3	−9.109	−9.885	−9.284
Delta	0.628	1.981	1.262
Rank	3	1	2

**Table 21:** ANOVA table for kerf taper angle response of input cutting parameters of 4 mm thickness of SPF-UPE composite cut with abrasive water jet technique.

Source	DF	Seq SS	Contribution	Adj SS	Adj MS	F-Value	P-Value
Water Pressure MPa	2	0.039044	4.33%	0.039044	0.019522	14.36	0.065
Traverse Speed mm/min	2	0.552278	61.20%	0.552278	0.276139	203.18	0.005
Stand-Off-Distance mm	2	0.308340	34.17%	0.308340	0.154170	113.44	0.009
Error	2	0.002718	0.30%	0.002718	0.001359		
Total	8	0.902379	100.00%				

**Table 22:** L9 array of input parameter levels with measured kerf taper angle θ° in degrees and calculated signal-to-noise (S/N) ratio based on the Taguchi method for 6 mm specimen thickness cut with abrasive water jet technique.

Ex No:	Water Pressure MPa	Traverse Speed mm	Stand-Off-Distance mm	Kerf Taper Angle θ°	SNRA1
1	300	1200	1	1.423	−3.06410
2	300	1400	2	1.762	−4.92012
3	300	1600	3	2.321	−7.31350
4	320	1200	2	1.912	−5.62976
5	320	1400	3	1.796	−5.08613
6	320	1600	1	1.980	−5.93330
7	340	1200	3	1.545	−3.77857
8	340	1400	1	1.698	−4.59875
9	340	1600	2	2.298	−7.22700

**Table 23:** S/N ratio response table of input cutting parameters of the 6 mm thickness of SPF-UPE composite cut with abrasive water jet technique.

Level	Water Pressure MPa	Traverse Speed mm	Stand-Off-Distance mm
1	−5.099	−4.157	−4.532
2	−5.550	−4.868	−5.926
3	−5.201	−6.825	−5.393
Delta	0.450	2.667	1.394
Rank	3	1	2

**Table 24:** ANOVA table for kerf taper angle response of input cutting parameters of 6 mm thickness of SPF-UPE composite cut with abrasive water jet technique.

Source	DF	Seq SS	Contribution	Adj SS	Adj MS	F-Value	P-Value
Water Pressure MPa	2	0.004436	2.06%	0.004436	0.002218	0.22	0.820
Traverse Speed mm/min	2	0.151712	70.35%	0.151712	0.075856	7.51	0.117
Stand-Off-Distance mm	2	0.039324	18.23%	0.039324	0.019662	1.95	0.339
Error	2	0.020191	9.36%	0.020191	0.010096		
Total	8	0.215664	100.00%				
